# New Zealand Nursing Service

**Published:** 1918-05-18

**Authors:** 


### NEW ZEALAND NURSING SERVICE.

THE New Zealand contingent of military nurses, which now numbers close on 500, had its beginning in August 1914, about the time that the earliest British nurses were arriving in Flanders. Six sisters and nurses, ignorant of their destination, embarked with the troops sent off on the declaration of war to find themselves, at the end of an adventurous voyage, in Samoa. This first group of nurses included Miss B. Nurse, R.R.C., Miss Fanny Wilson, A.R.R.C., Miss Beda Maclean, A.R.R.C., Miss L. McNie, A.R.R.C., Matron E. G. Brooke, R.R.C. After the speedy rounding up of the Samoan Islands, the sisters were sent on to Egypt, where thirty-five New Zealand nurses are still working, attached to Imperial hospitals. At first a New Zealand Hospital was taken over by the military authorities to form a training ground and base for the Military Service, but two years ago the headquarters of the New Zealand Nursing Service were moved to London, where, from her office in Southampton Row, their able Matron-in-Chief, Miss Thurstan, controls her staff in their various centres of activity. Among the establishments which are staffed by New Zealand nurses are the No. 1 New Zealand Hospital at Brockenhurst, the No. 2 N.Z.H. at Walton-on-Thames, each containing over 1,000 beds, and the No. 3 N.Z.H. at Codford on Salisbury Plain, which, with its venereal section, makes up another 1,000 beds. At Hornchurch in Essex there is a New Zealand Convalescent Hospital and camp, with 100 beds and 2,000 camp beds. It has massage and electrical departments and is admirably equipped for its purpose. The Service also staffs a stationary hospital in France with 500 beds, now made up to 1,000. Some idea of the vicissitudes of nursing in France may be gathered from the fact that this hospital has moved out three times.

The nurses are all engaged for the duration of the war. The staff nurses receive £100 a year, the sisters £120, matrons £150, the latter reckoned very low pay according to the rates prevailing in civil hospitals in New Zealand, where the matrons of the four large hospitals start at £230, in addition to liberal allowances. On receiving her appointment, each nurse receives an equipment allowance of £15 15s., the former rate of £10 10s. having been found insufficient. Besides this they get a grant for camp kit of £8. The uniform is grey with scarlet facings, very neat and effective. The coat has brass buttons stamped N.Z., with the Southern Cross. Nurses are allowed three weeks' leave in the year, and a day once a month. There is a charming little hospital and convalescent home at Brighton with forty beds for the use of nurses who are ill or run down. But the health of the Force has been well maintained since their arrival. Since the terrible catastrophe in the Ægean Sea, when ten nurses went down in the *Marquetta*, there have been no accidents in coming or going. Owing to the fact that members of the Service are nearly all known to each other and to their superior officers, there is a strong spirit of union among them. Discipline is undoubtedly easier to maintain under these circumstances, and New Zealand has every reason to be very proud of the women they have sent over to play their part in this bitter struggle.

☞ For Women's Work in the War, see p. 148; Matrons' and Sisters' Appointments, p. iv.

